# Estimated Daily Intake and Cumulative Risk Assessment of Phthalates in the General Taiwanese after the 2011 DEHP Food Scandal

**DOI:** 10.1038/srep45009

**Published:** 2017-03-22

**Authors:** Jung-Wei Chang, Ching-Chang Lee, Wen-Harn Pan, Wei-Chun Chou, Han-Bin Huang, Hung-Che Chiang, Po-Chin Huang

**Affiliations:** 1 Research Center for Environmental Trace Toxic Substances, National Cheng Kung University, Tainan, Taiwan; 2 Department of Environmental and Occupational Health, College of Medicine, National Cheng Kung University, Tainan, Taiwan; 3 Institute of Biomedical Sciences, Academia Sinica, Taipei, Taiwan; 4 Division of Preventive Medicine and Health Service Research, Institute of Population Health Sciences, National Health Research Institutes, Miaoli, Taiwan; 5 National Environmental Health Research Center, National Institute of Environmental Health Sciences, National Health Research Institutes, Miaoli, Taiwan; 6 School of Public Health, National Defense Medical Center, Taipei, Taiwan; 7 Research Center for Environmental Medicine, Kaohsiung Medical University, Kaohsiung, Taiwan; 8 Department of Safety, Health and Environmental Engineering, National United University, Miaoli, Taiwan

## Abstract

A food scandal occurred in Taiwan in 2011 because the DEHP (di-2-ethylhexyl phthalate) had been intentionally used in food products. We assessed the daily intakes (DIs) and cumulative risk of phthalates in Taiwan’s general population after the scandal. The DIs of 6 phthalates, including di-n-butyl phthalate (DnBP), di-iso-butyl phthalate (DiBP), and DEHP, were evaluated using urinary phthalate metabolites. Hazard quotients of phthalates classified as affecting the reproductive (HQ_rep_) and hepatic (HQ_hep_) systems were assessed using cumulative approach. The creatinine-based model showed that the highest DI values in children 7-to 12- years-old were for DEHP (males: median: 4.79 μg/kg bw/d; females: median: 2.62 μg/kg bw/d). The 95^th^ percentile (P95) of HQ_rep_ values were all >1 in the 7- to 12-year-old and 18- to 40-year-old male groups. The P95 of HQ_hep_ values were all >1 in the 7- to 18- year-old male groups. Most of the HQ_rep_ was attributable to the HQs of DnBP and DiBP (53.9–84.7%), and DEHP contributed most to HQ_hep_ (83.1–98.6%), which reveals that DnBP, DiBP and DEHP were the main risk of phthalate exposure for Taiwanese. Taiwan’s general population is widely exposed to DnBP, DiBP and DEHP, especially for young children.

Phthalates are synthetic aromatic chemicals adding in a variety of products used in everyday life^1^,^2^. Di-(2-ethylhexyl) phthalate (DEHP), di-n-octyl phthalate (DnOP), di-isononyl phthalate (DiNP), and diisodecyl phthalate (DiDP) belongs to the high-molecular-weight phthalates and are used as plasticizers in building materials and furniture, and di-*n*-butyl phthalate (DnBP), di-*i*-butyl phthalate (DiBP), dimethyl phthalate (DMP). and diethyl phthalate (DEP) are used in personal-care products (e.g., nail polish, fragrance, etc.), lacquers, varnishes, and coatings for their nature of low molecular weight^3^. The major phthalates exposure route for the general population are ingestion, inhalation, and dermal absorption^4^,^5^,^6^. Dietary exposure is believed to be the major important source for the general population^7^,^8^,^9^. Additionally, young children could be exposed to phthalates when they swallow or inhale dust while playing on the floor, and by chewing PVC containing toys and products^10^,^11^. The phthalate metabolites have been measured in a representative U.S. general population urinary samples^12^, in some European countries^13^,^14^,^15^ and Asian countries^16^,^17^ have also been reported, which indicates that phthalates exposure can happen anywhere, at any time.

Based on human-biomonitoring data, the risk assessment of different phthalates can be individually evaluated. Human-biomonitoring is a method used to approximate the total background exposure of phthalic acid esters (PAEs). Furthermore, based on the hazard index (HI) approach^18^,^19^, which assumes dose addition^20^,^21^, a cumulative risk assessment for various exposures to chemicals with similar toxicity has been developed for PAEs. As outlined in a National Research Council (NRC) report (2008), the HI provides a direct and simple method for relating the intake of a certain of chemicals to their reference values (RfVs). Examples of RfVs for oral exposure include the U.S. EPA reference doses (RfDs)^21^, and the European Food Safety Authority (EFSA) tolerable daily intake (TDI)^22^,^23^,^24^,^25^. The hazard quotient (HQ) is estimated as the ratio of the calculating exposure level to the RfV of corresponding chemical. The HQs are then added together to estimate the overall HI.

After the 2011 Taiwan DEHP scandal, the Taiwan Food and Drug Administration (TFDA) adopted the EFSA TDI values. It is difficult to know whether the current reference values protect general Taiwanese. Several parameters of risk assessment of PAEs vary widely based on ethnicity^26^,^27^. Therefore, we used human-biomonitoring data from our published study to estimate the daily exposure dose and cumulative risk of phthalate in Taiwan’s general population. Additionally, we also try to identify potential differences and trends in exposure to phthalates. The objectives of this study were: (1) to estimate the daily intakes (DIs) of six phthalates based on their urinary levels; and (2) to assess the cumulative risk of exposure to phthalates based on anti-androgenic and hepatic endpoints in a Taiwanese general population.

## Results

The detectable rate of phthalate metabolites was highest in MEHHP and lowest in MiNP in all urine samples. Geometric means were from ND to 32.7 mg/L for the 10 phthalate metabolites measured. The median level of MEP (creatinine-unadjusted) in the 18–40 years old group was significantly (p < 0.001) higher than that in the other age groups, especially for women. Additionally, median levels of MnBP and MiBP in all participants decreased along with increasing age. There was no significant change of MEHP in our participants regardless of age or gender.

### Estimated daily intake of phthalates

The risks associated with exposure to phthalates were assessed based on the DI of each participant (Table 1). The DI was compared with the EFSA TDI acceptable exposure level in order to estimate potential exceedances. The DIs of DnBP, DiBP, and DEHP decreased with increasing age. Median DIs of DnBP, DiBP, and DEHP were nearly four times as high in the ≥7–<12 years old than in the >65 years old males group. The DIs of DiBP and DEHP for the ≥7–<12 years old females group were all slightly higher than those for the other female age groups. The DIs of DEHP for males were all slightly higher than those for females in all age groups. The creatinine-based calculation model showed that the highest DI values in males and females ≥7–<12 years old were for DEHP (males: median: 4.79 μg/kg bw/d; 95^th^ percentile (P95): 22.6 μg/kg bw/d; females: median: 2.62 μg/kg bw/d; P95: 12.6 μg/kg bw/d). DEHP also had the highest DI values in young adult (≥18–<40 years old) males and females (males: median: 4.03 μg/kg bw/d; P95: 12.1 μg/kg bw/d; females: 2.31 μg/kg bw/d; P95: 18.2 μg/kg bw/d). DIs for BBzP and DiNP were considerably lower in all age-gender groups, but within the same range (median: 0. 005–0.252 μg/kg bw/day).

### Reproductive and hepatic evaluation. Cumulative risk assessment: HQ and HI

**Figure 1:**
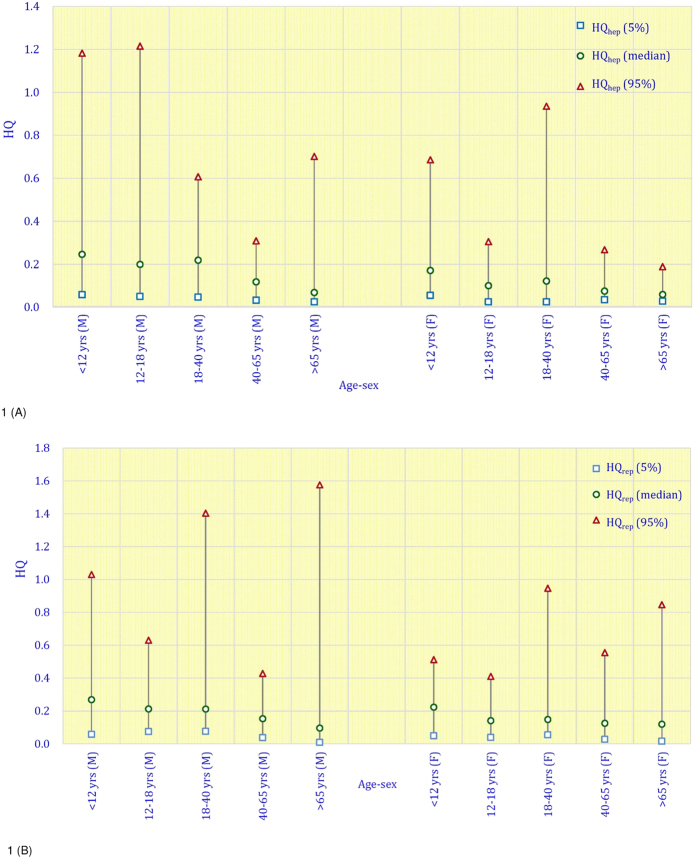
The median and 95th percentile hazard quotient (HQ) for (**A**) hepatic effects; and for (**B**) anti-androgenic effects of four phthalates in different age groups by gender based on the EFSA TDIs and USEPA RfD.

More males had higher HQ_rep_ values: HQ_rep_ > 1: males = 2.66%; females = 2.01% (Fig. 1). The P95 of HQ_rep_ values were all >1 in the ≥7–<12-year-old, 18- to 40-year-old, and ≥65-year-old male groups, but they were <1 in all of the female groups. More males had higher HQ_hep_ values: HQ_hep_ > 1: males = 2.13%; females = 0.50%. The P95 of HQ_hep_ values were all > 1 in the ≥7–< 12-year-old and 12- to 18- year-old male groups, but they were <1 in all of the female groups.

Except for 18- to 40-year-olds, in the other age groups, HQ_hep_ values were non-significantly higher in males than in females; however, in the ≥40- to <65-year-old group, the difference was significant (p = 0.001) (Fig. 1). Although median values of HQ_hep_ were comparable in males and females, the P95 was about twice as high for males than for females in the ≥7–<12-year-old, ≥18- to <40-year-old, and >65-year-old age groups. Differences in gender were particularly pronounced in the ≥7–≤12 years old, and ≥12- to <18 years old groups. In addition, HQ_rep_ values in males were also slightly higher than in females, except for the ≥18- to <40-year-olds (Fig. 1). Although the median values of HQ_rep_ were comparable in males and females, the P95 was about twice as high for males than for females in the ≥7–<12 and >65 years old groups. Differences in gender were particularly pronounced in the children and in the elderly.

**Figure 2:**
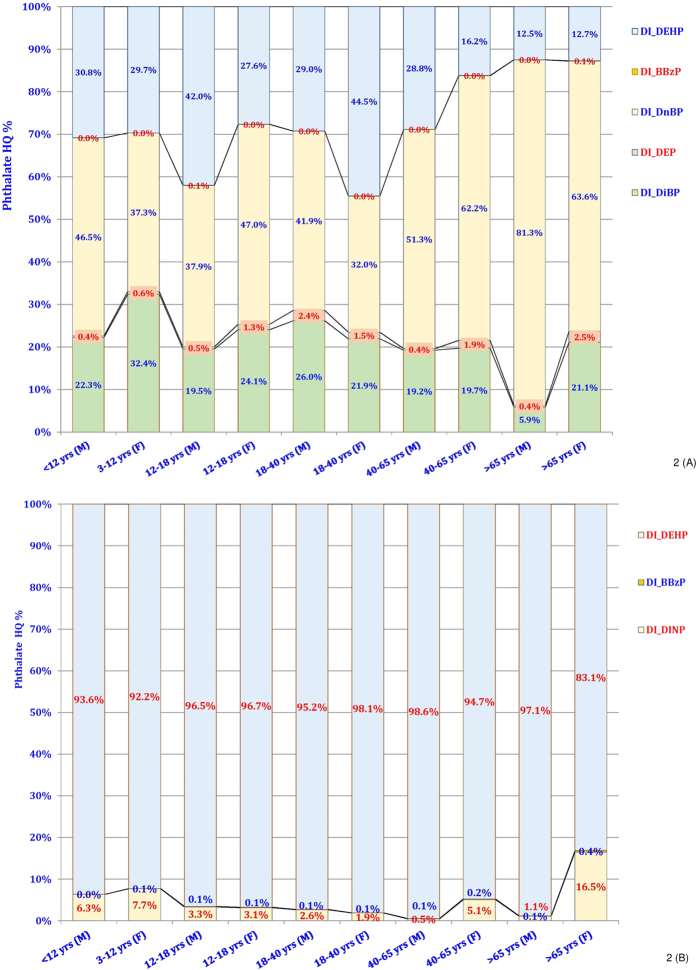
The contribution of different PAEs to hazard quotient (HQ) for (**A**) hepatic effects; and for (**B**) anti-androgenic effects in different age groups by gender.

In general, most of the HQ_rep_ was attributable to the HQs of DnBP and DiBP (53.9–84.7%), and DnBP and DiBP combined was the main risk of phthalate exposure for Taiwanese adults (Fig. 2). DEHP contributed most to HQ_hep_ (83.1–98.6%), which might have been because of the higher DEHP exposure dose and hepatic toxicity than of the other two phthalates (Fig. 2).

**Figure 3:**
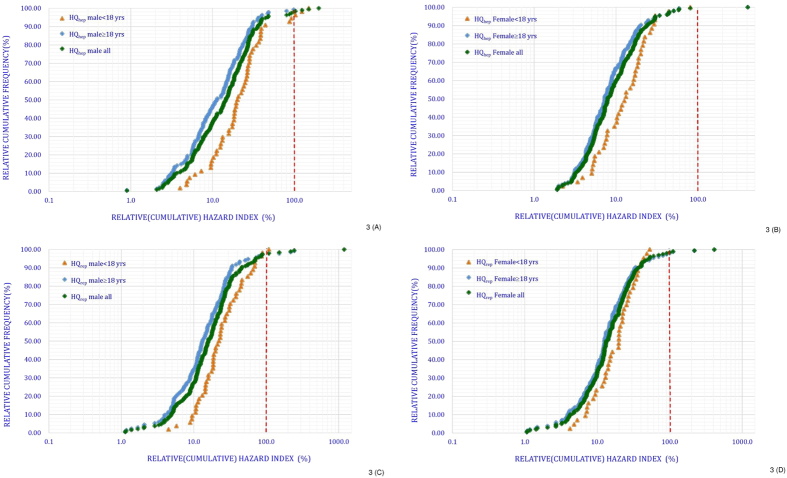
Cumulative risk of hepatic (**A**,**B**) and anti-androgenic effects (**C**,**D**) of phthalates in adults and minors: The dotted 100%-line illustrates the HI in respect to the cumulative HI (HIcum) for the different scenarios.

Figure 3 illustrates the distribution plots of HQ_hep_ and HQ_rep_ (logarithmic scale for the *x*-axis) against the relative cumulative frequency distribution, which must be seen as a first crude approach for determining the cumulative exposure and additive toxicity of phthalates. Depending upon the cumulative model, 4 males (3 were <18 years old [5.6%]; 1 was ≥18 years old [0.75%]) and 1 female (≥18 years old [0.64%]) had high (>1) HQ_hep_ values. In addition, 5 males (1 was <18 years old [1.9%]; 4 were ≥18 years old [2.99%]) and 4 females (≥18 years old [2.56%]) had high (>1) HQ_rep_ values, which indicated a considerable level of cumulative exposure to phthalates for Taiwanese adults.

### Principal Component Analysis (PCA)

**Figure 4:**
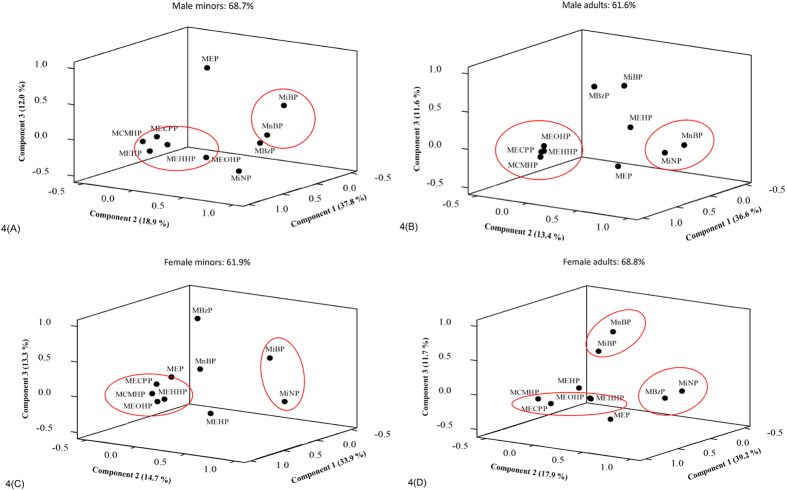
Principal component analysis of 10 urinary phthalate metabolites (minors (**A**,**C**) and adults (**B**,**D**)) by gender (male (**A**,**B**) and female (**C**,**D**)).

**Figure 5:**
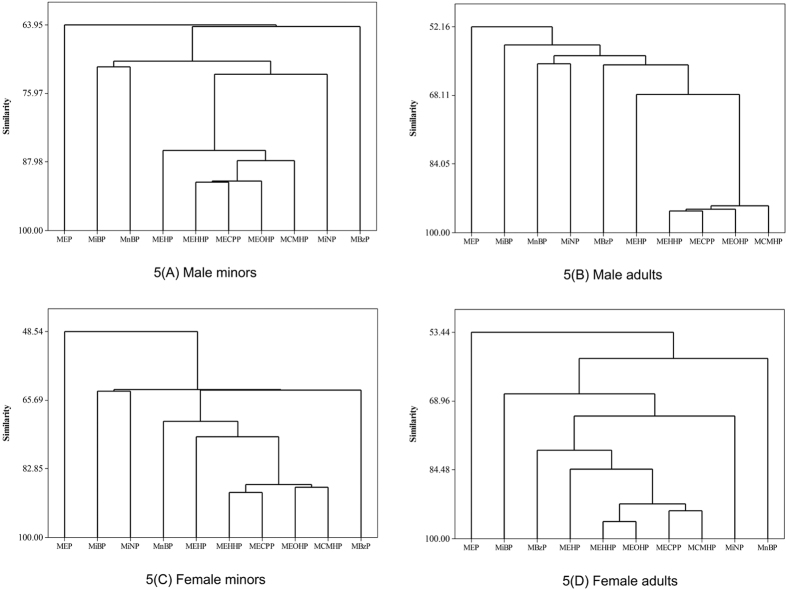
Cluster analysis of 10 urinary phthalate metabolites (minors (**A**,**C**) and adults (**B**,**D**)) by gender (male (**A**,**B**) and female (**C**,**D**)).

To evaluate the exposure profile of phthalates in different age and gender, the PCA results are divided into male minors (<18 years old), male adults (≥18 years old), female minors, and female adults, and described in Fig. 4 and Supplementary Table 2. Three principal components were extracted from male minors, which accounted for 37.8% (Principal Component 1 [PC1]), 18.9% (PC2), and 12.0% (PC3) of the variability. This indicated three major potential sources of exposure to phthalates for Taiwanese male minors. The 5 DEHP metabolites—MEHP, MEHHP, MEOHP, MECPP, and MCMHP—were highly correlated with PC1, and MiBP and MnBP were highly correlated with PC2. Three principal components were extracted from male adults, which accounted for 36.6% (PC1), 13.4% (PC2), and 11.6% (PC3) of the variability. Four DEHP metabolites—MEHHP, MEOHP, MECPP, and MCMHP—were highly correlated with PC1, and MnBP and MiNP were highly correlated with PC2. Three principal components were extracted from female minors, which accounted for 33.9% (PC1), 14.7% (PC2), and 13.3% (PC3) of the variability. The 4 DEHP metabolites—MEHHP, MEOHP, MECPP, and MCMHP—were highly correlated with PC1, and MiBP and MiNP were highly correlated with PC2. Three principal components were extracted from female adults, which accounted for 39.2% (PC1), 17.9% (PC2), and 11.7% (PC3) of the variability. The 4 DEHP metabolites—MEHHP, MEOHP, MECPP, and MCMHP—were highly correlated with PC1, and MBzP and MiNP were highly correlated with PC2. In addition, MiBP and MnBP were highly correlated with PC3. Moreover, in male minors, our cluster analysis (CA) identified two major clusters of urinary metabolites: (1) the DEHP metabolites (MEHP, MEHHP, MEOHP, MECPP, and MCMHP); and (2) MiBP and MnBP (Fig. 5). In male adults, CA identified two major clusters of urinary metabolites: (1) the DEHP metabolites (MEHHP, MEOHP, MECPP, and MCMHP); and (2) MnBP and MiNP. In female minors, CA identified two major clusters of urinary metabolites: (1) the DEHP metabolites (MEHHP, MEOHP, MECPP, and MCMHP); and (2) MiBP and MiNP. In female adults, CA identified three major clusters of urinary metabolites: (1) the DEHP metabolites (MEHHP, MEOHP, MECPP, and MCMHP); (2) MBzP and MiNP; and (3) MiBP and MnBP.

We used information from the questionnaire to evaluate the relationships between urinary phthalate metabolites, phthalate DIs, and family members (Supplemental Table 3). The correlation coefficients (*r*) of urinary MiBP, MnBP, MEHHP, MEOHP, MECPP, MCMHP, and ΣDEHP were higher in couples and siblings than in parents. Moreover, the *r* of DiBP, DnBP, DEP, and ΣDEHP DIs were also higher in couples and siblings than in parents.

## Discussion

In the present study, we systematically measured our study sample’s daily exposure to phthalates based on biomonitoring data, and provide a comprehensive and cumulative estimated risk of exposure to phthalates for Taiwan’s general population, the first study to do so.

Our human-biomonitoring data are measurements of internal doses from all routes (inhalation, dermal, and oral) and sources of exposure to phthalates, and provide an effective tool for assessing the general population’s exposure. Most assessments of exposure to phthalates in currently focused on human-biomonitoring^2^,^28^,^29^. The National Health and Nutrition Examination Survey (NHANES) data have been used to back-calculate daily exposure to chemicals in the US general population^30^,^31^. In a recent study, reverse dosimetry approach were used to reconstruct exposure from urinary concentrations of 82 NHANES chemicals involving phthalate compounds to prioritize their risk^32^. Hays and colleagues developed the biomonitoring equivalents to evaluate the risk from urine data for DiNP^33^. Several studies have reconstructed daily does from phthalate or its metabolites in urine for comparison to the existing RfD^34^,^35^. These studies show that exposure reconstruction from phthalate and its metabolites in urine are regarded as the most reliable method to quantify overall exposure to phthalates because phthalate metabolites in urine are not likely to have bias by external contamination.

The results generated by dose addition models were consistent with actual administration experiments of several phthalates simultaneously^36^,^37^,^38^,^39^,^40^. To protect the public’s health, the TDI was used to estimate the amount of a chemical in air, food, and drinking water that can be consumed everyday over a lifetime without appreciable health risk. In the present study, the maximum and P95 values for DnBP and DEHP in male minors group were very close the TDI reference values, which indicates that the latter for some individual PAEs are recommended to be modified. Therefore, we can also protect the most vulnerable segments of the general population from exposure to PAEs. In Taiwan, there is no suggested TDI value for DiBP, even though this phthalate was reported^41^ to reduce fetal testosterone production with a potency similar to that of both DnBP and DEHP, which suggests that it is appropriate to use the EFSA TDI value for DnBP to estimate exposure to DnBP and DiBP simultaneously. For vulnerable groups—especially children—the growing evidence indicated that growth and development of the reproductive and endocrine systems could be disrupted^42^,^43^,^44^,^45^. In addition, results from the recent toxicological phthalates studies have indicated a consideration of each TDI for each individual phthalate would be inappropriate for the overall tolerable phthalate intake^38^,^39^,^40^. The omnipresent exposure to a lot of phthalates and the understanding that these phthalates act in a dose-additive nature derived a cumulative risk assessment method^38^,^39^,^40^.

We used a set of equations developed by Mage *et al*.^26^,^27^ to predict our participants’ expected daily creatinine excretion (CE) (mg/kg) as a function of age, gender, and anthropometric measurements, in two age groups (≥7–18 and ≥18 years old). We also compared estimated DIs using the CE equations separately developed by Mage *et al*.^26^,^27^ and Kawasaki *et al*.^46^. Although the Mage *et al*. CE equation yields a lower DI, we still use it because it considers different physiological parameters for each age group, especially for minors. The HQ and HI approaches provide a forthward method to evaluate non-cancer risk for a given level of chemical exposure.

Calculating and interpreting the HQ and HI depend upon the method used to estimate level of exposure, and on the choice of a reference value. The creatinine correction approach evaluated by Aylward *et al*.^47^, assumes that a sampled concentration sufficiently represents a daily average concentration. Thus, DIs derived from spot samples might range widely of the actual DI (20% to 300%), although this variability might affect the accuracy of an estimated intake for a single individual. A group of spot urine samples provides a reasonable approximation of concentrations that could have been observed in a population of full-day urine samples collected from the same population for phthalates^48^,^49^.

Principal component analysis (PCA) was used to characterize the similarity of urinary phthalate metabolites in each participant. Cluster analysis yielded similar findings. We found that the primary component of exposure to PAEs in Taiwanese was DEHP, regardless of age or gender. Guo *et al*.^50^ found that DEHP was the most abundant phthalate in food in a Chinese population. Due to a similarity of life style and habit of food consumption in Han population, food might also be the major source of exposure to DEHP for the Taiwan general population. The secondary components of exposure to PAEs in Taiwanese were MiBP, MnBP, MBzP, and MiNP. The sources of DiBP, DnBP, BBzP, and DiNP were more complicated. Guo *et al*. and Guo and Kannan claimed that diet, dust, and personal care products were not major sources of exposure to DnBP and DiBP for the Chinese general population^50^,^51^,^52^. We previously found that beverages were the primary contributors (about 30–60%) in the overall estimates of average daily doses (ADDs) for all PAEs^53^. This might raise public concern, because the health of some Taiwanese (children in particular) is probably being negatively affected because they drink too much artificially sweetened tea and other soft drinks from phthalate-containing plastic cups and bottles. We also found that the P95 HIs of anti-androgenic phthalates in children were already >1.

Despite the claims of Guo *et al*. and Guo and Kannan^50^,^51^,^52^, others^51^,^54^,^55^ concluded that personal care products application were the major sources of DEP and DnBP in the environment. Parlett *et al*. and Philippat *et al*. documented that increased phthalate levels in women were correlated with using greater amounts of cosmetic, perfume, and personal care products^56^,^57^. We previously found that only 45% of Taiwanese women used lotion and body wash every day, and that less than 5% of them used perfume and nail polish frequently^58^. Therefore, personal care products might be another source of exposure to phthalates for the Taiwan general population. Moreover, the correlation between urinary phthalate metabolites and phthalate DIs of DiBP, DnBP, DEP, and ΣDEHP in couples and siblings are higher than in their parents. This probably indicates that cohabiting adults are more similar in their levels of exposure to PAE than are parents and the children they are raising, or that the older people become, the fewer phthalate-containing personal care products they use.

This study has several strengths. First, it is a nationwide sample with participants between 7 and 97 years old groups. These data provide reference values of phthalates in the Taiwanese general population. Second, we use an appropriate equation that estimates daily creatinine excretion (mg/day) based on several physiological parameters. This developed equation is piecewise continuous for males and females from 7 to 97 years old. Third, we determined the internal exposure to phthalates in the general population and used it to estimate phthalate-related health risks. This study has some limitations. First, the distribution of individual exposure to phthalates in a general Taiwanese population after a DEHP food scandal can vary and the cross-sectional study design with one-time-point measurements cannot sufficiently assess exposure over time. The estimation of phthalate DIs is based on urinary metabolite levels measured in first morning samples and on the premise that the concentrations of metabolites in these spot morning samples are representative of the daily average urinary levels. Second, we did not have sufficient evidence to link decreased phthalate exposure to this DEHP food scandal. Legal restrictions on the products in which phthalates can be used, and the permissible levels of phthalates might decrease phthalate exposure levels in the general population.

## Conclusion

We assessed the DIs and cumulative risks of 6 phthalates in a Taiwan population based on the urinary phthalate metabolites after the 2011 DEHP food scandal. Our data indicated that the Taiwanese general population is still widely exposed to phthalates after restrictions and legislation on phthalates, and showed that HQ_hep_ and HQ_rep_ values were slightly higher in males than in females, especially for 18- to 40-year-olds. We also found two components of exposure to PAEs: the primary was DEHP and the secondary were DiBP, DnBP, and BBzP. Additional studies are needed to clarify whether the contamination sources primarily food and personal care products. We suggest that Taiwan’s government lower the TDI of DEHP to protect vulnerable residents (children and adults of childbearing age) after considering the potential cumulative negative effects on reproduction.

## Materials and Methods

### Study Participants and Biomonitoring Data of Phthalates in Human Urine Samples

The subjects recruiting and sampling process were approved by the Research Ethic Committee of National Health Research Institutes (No. EC1020206) and described elsewhere^58^. The methods were performed in accordance with the approved guidelines.

After a written informed consent on behalf of the participated children was obtained from their parents and each child, participants provided a first morning urine sample and filled in data on a systematic questionnaire about participant demographics (age, gender, body weight, body height, and residence). The participants recruited consisted of 199 females and 188 males living in 22 cities and counties in Taiwan. The participants were between 7 and 97 years old, with an average body mass index (BMI) of 23.5 kg/m^2^. Immediately after the May-to-December 2013 urine collection, the samples were aliquoted, stored at −80 °C and thawed at −20 °C before sample pretreatment. Therefore, the urinary levels of phthalate metabolites were measured in the following participants: children (≥7–<12 years old), adolescents (≥12–<18 years), young adults (≥18–40 years), middle-aged adults (≥40–<65 years), and the elderly (≥65 years) were analyzed and used to calculate DIs of PAEs and to estimate cumulative risks. To calculate the DIs of PAEs and then the hazard index (HI), which was recently established for assessing the cumulative risk of phthalates exposure^19^,^59^,^60^, we used the results of urinary levels of PAE metabolites determined in our previous study^58^. By considering the cumulative hazard of several phthalates with similar toxicity, this index was evaluated by adding the ratios between TDI and reference limits (TDI or RfD anti-androgenicity) for the different compounds. The first-morning urine samples were analyzed for ten phthalate metabolites [mono-ethylhexyl phthalate (MEHP), mono-(2-ethyl-5-oxo-hexyl) phthalate (MEOHP), mono-(2-ethyl-5-hydroxyhexyl) phthalate (MEHHP), mono-(2-ethyl-5-carboxypentyl) phthalate (MECPP), mono-(2-carboxymethylhexyl) phthalate (MCMHP), mono-n-butyl phthalate (MnBP), mono-iso-butyl phthalate (MiBP), monoethyl phthalate (MEP), mono-iso-nonyl phthalate (MiNP), and mono-benzyl phthalate (MBzP)] which are biomarkers for exposure to the six commonly used phthalates [DEHP, DnBP, DiBP, DEP, DiNP, and benzyl butyl phthalate (BBzP)]. We used an online modified analytical method coupled to a liquid chromatograph/electrospray tandem mass spectrometer (Agilent 1200/API 4000; Applied Biosystems, Foster City, CA, USA) method discussed by Koch *et al*.^61^,^62^ with quantification by isotope dilution.

### Calculating Daily Intakes (DIs)

To calculate the DIs of each phthalate, the urinary phthalate metabolite levels in the spot urine samples and the individual age, body weight (BW), and body height (ht) data of each participant were combined. The individual DIs of phthalates based on urinary phthalate metabolites were calculated using the method described by Koch *et al*.^63^:


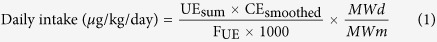

1. UE_sum_ is the molar urinary excretion sum of the measured urinary phthalate metabolites;
2. The smoothed creatinine excretion (CE) rates CE_smoothed_ are age, body weight (BW) and height (ht), and gender-based values for urinary CE^26^,^27^. The formulae of CE_smoothed_ estimates for adults and minors in this study are listed below:
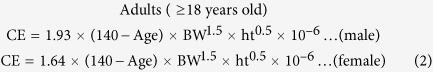

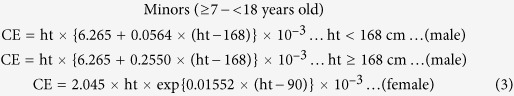
3. where Age (years old) and ht (cm) are the participant’s age and height, which were obtained from the questionnaire;
4. F_UE_, the molar fraction, describes the molar ratio between the excreted amounts of the specific metabolites of each phthalate corresponding to the dietary intake of the parent phthalate^2^,^64^.

All of the parameters we used for calculating DIs are listed in Supplemental Table 1.

### Cumulative Risk Assessment — Hazard Quotient and Hazard Index

To assess the participant’s risk from each phthalate, we used the hazard quotient (HQ) as following formula:


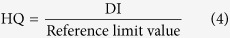


Where HQ is the hazard quotient for an individual phthalate, the reference limit value (RLV) is the TDI or RfD. The RLV selected for DnBP, BBzP and DEHP by the EFSA^22^,^23^,^24^ were 10, 500 and 50 μg/kg-BW/day, and RfDs developed for BBzP and DEHP by the U.S. EPA^65^,^66^ were 200 and 20 μg/kg-BW/day. There was no TDI or RfD for DiBP; thus, the DnBP value was based on analogy assignment, 10 μg/kg-BW/day^19^,^59^. The TDI values of DnBP, DEHP, and BBzP set up by the EFSA were based on anti-androgenic effects (developmental and testicular toxicity) in animal models^22^,^23^,^24^.

An HI < 1 indicates no significant adverse effects from several chemicals exposure could happen^67^. Estimated HI values of cumulative hepatic effect were calculated based on the RfD (HQ of BBzP, DiNP, and DEHP).


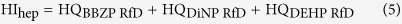


Because not all of the U.S. EPA’s RfDs for phthalates were established based on anti-androgenic effects, HI values were estimated based only on the TDIs (HQ of DnBP, DiBP, BBzP, and DEHP). Although the TDI and RfD for DiBP could not be obtained, it was assuming DiBP with the values of DnBP for their similar structure and toxicity^36^. DEP is not included in the anti-androgenic assessment for its corresponding toxicity^68^.


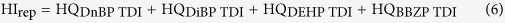


We calculated the DIs for each phthalate and compare them to the EFSA’s TDIs; normalized each TDI to 100%; summed the DI percentages calculated in relation to the respective TDIs; and then checked to see whether the cumulative TDI (at 100%) had been exceeded.

### Principle Component Analysis

We used principle component analysis (PCA) for 10 phthalate metabolites (MEP, MBzP, MnBP, MiBP, MiNP, and the five DEHP metabolites) to identify potential sources. The number of components to retain was based on score plot analysis and eigenvalue [1.1] criteria. We use varimax (orthogonal) rotation to obtain a set of independent interpretable factors according to a factor loading >0.55 (or <−0.55) with a particular factor are considered to be its major constituents. In addition, an agglomerative hierarchical clustering analysis was used to group the phthalate metabolites into clusters.

### Statistical Analysis

We report phthalate results as μg/g of creatinine (μg/g Cr). Creatinine was used to adjust for individual variations in urine concentration. The non-detectable (ND) levels, i.e., those below the limit of detection, were calculated as half of the detection limit of each phthalate metabolite, and the detectable rate as the number of urine samples with the level of each phthalate metabolite above the detection limit, divided by all of the analyzed urine samples. We categorized our participants into five comparably sized age groups: ≥7–<12 years old, ≥12 to <18 years old, ≥18 to <40 years old, ≥40 to <65 years old, and ≥65 years. The Mann-Whitney U test was used to evaluate differences between demographic data, e.g., age and gender, and the Kruskal-Wallis test was used to evaluate differences between each level of phthalate metabolites. SPSS 22.0 (SAS Institute, Cary, NC, USA) for Windows was used for all statistical analyses. Significance was set at p < 0.05.

## Additional Information

**How to cite this article:** Chang, J.-W. *et al*. Estimated Daily Intake and Cumulative Risk Assessment of Phthalates in the General Taiwanese after the 2011 DEHP Food Scandal. *Sci. Rep.* **7**, 45009; doi: 10.1038/srep45009 (2017).

**Publisher's note:** Springer Nature remains neutral with regard to jurisdictional claims in published maps and institutional affiliations.

## Supplementary Material

Supplementary Tables

## Figures and Tables

**Table 1:** Estimated DEP, DiBP, DnBP, BBzP, DiNP, and DEHP daily intake (μg/kg body-weight/day) and hazard quotient (HQ) calculated for each age-sex group.

Phthalate		DEP	DiBP	DnBP	BBzP	DiNP	DEHP
Male/<12 yrs	DI	0.043 (0.007–6.12)^b^	0.619 (0.002–5.07)	1.12 (0.002–7.81)	0.005 (0.002–0.149)	0.210 (0.087–66.2)	4.79 (0.862–24.8)
(n = 25)	HQ^a^	0.0008 (0.00001–0.01)	0.06 (0.0002–0.51)	0.112 (0.0002–0.781)	0.00001 (0.000004–0.0003)	0.0014 (0.0006–0.441)	0.095 (0.017–0.495)
Male/12–18 yrs	DI	0.423 (0.004–2.94)	0.472 (0.003–1.37)	0.688 (0.003–2.53)	0.04 (0.001–0.411)	0.181 (0.051–14.0)	3.88 (0.778–29.7)
(n = 29)	HQ^a^	0.0009 (0.000009–0.006)	0.05 (0.0003–0.14)	0.07 (0.0003–0.253)	0.00008 (0.00003–0.0008)	0.0012 (0.0004–0.0935)	0.078 (0.016–0.59)
Male/18–40 yrs	DI	0.856 (0.08–87.1)	0.434 (0.003–5.42)	0.581 (0.004–19.6)	0.006 (0.001–0.655)	0.181 (0.04–18.3)	4.03 (0.317–16.0)
(n = 31)	HQ^a^	0.002 (0.0002–0.17)	0.043 (0.003–0.542)	0.058 (0.0004–1.96)	0.00001 (0.000002–0.001)	0.0012 (0.0002–0.122)	0.08 (0.0063–0.321)
Male/40–65 yrs	DI	0.273 (0.002–10.4)	0.246 (0.003–2.41)	0.521 (0.003–13.9)	0.005 (0.002–0.269)	0.172 (0.066–3.46)	2.23 (0.132–9.63)
(n = 55)	HQ^a^	0.0005 (0.000003–0.02)	0.024 (0.0003–0.241)	0.052 (0.0003–1.39)	0.00001 (0.000003–0.0005)	0.0012 (0.0004–0.024)	0.046 (0.026–0.193)
Male/>65 yrs	DI	0.23 (0.003–11.2)	0.16 (0.003–1.81)	0.275 (0.003–118)	0.007 (0.002–0.288)	0.233 (0.068–4.79)	1.16 (0.376–39.2)
(n = 48)	HQ^a^	0.0005 (0.000007–0.02)	0.02 (0.0003–0.181)	0.028 (0.0004–11.8)	0.00001 (0.000003–0.0006)	0.002 (0.00045–0.032)	0.023 (0.008–0.785)
Female/<12 yrs	DI	0.28 (0.007–7.68)	0.489 (0.002–3.07)	0.785 (0.173–2.70)	0.007 (0.002–0.182)	0.252 (0.077–20.1)	2.62 (0.977–14.6)
(n = 26)	HQ^a^	0.0006 (0.00001–0.02)	0.05 (0.0002–0.31)	0.079 (0.0173–0.27)	0.00001 (0.000004–0.0004)	0.0017 (0.0005–0.134)	0.052 (0.0195–0.292)
Female/12–18 yrs	DI	0.549 (0.008–8.52)	0.248 (0.004–1.59)	0.646 (0.006–2.95)	0.005 (0.002–0.327)	0.215 (0.095–5.10)	1.87 (0.416–6.01)
(n = 17)	HQ^a^	0.001 (0.0002–0.02)	0.02 (0.0004–0.16)	0.065 (0.0006–0.295)	0.00001 (0.000005–0.0006)	0.0014 (0.0006–0.034)	0.037 (0.008–0.12)
Female/18–40 yrs	DI	0.581 (0.067–23.9)	0.319 (0.002–3.81)	0.624 (0.003–3.31)	0.006 (0.002–0.278)	0.197 (0.062–3.88)	2.31 (0.262–81.5)
(n = 37)	HQ^a^	0.002 (0.0001–0.05)	0.03 (0.0002–0.381)	0.062 (0.0003–0.33)	0.00001 (0.000003–0.0006)	0.0013 (0.0004–0.0259)	0.046 (0.0053–1.63)
Female/40–65 yrs	DI	0.435 (0.003–53.5)	0.3 (0.001–3.24)	0.476 (0.005–40.1)	0.006 (0.001–0.873)	0.233 (0.061–10.6)	1.33 (0.329–11.3)
(n = 74)	HQ^a^	0.0009 (0.000005–0.11)	0.03 (0.0001–0.324)	0.048 (0.0005–4.01)	0.000012 (0.000002–0.0017)	0.0016 (0.0004–0.07)	0.026 (0.007–0.226)
Female/>65 yrs	DI	0.284 (0.004–76.5)	0.16 (0.004–2.28)	0.554 (0.003–10.1)	0.006 (0.001–1.48)	0.25 (0.045–27.0)	1.04 (0.298–3.44)
(n = 45)	HQ^a^	0.0006 (0.000008–0.15)	0.016 (0.0005–0.228)	0.055 (0.0002–1.01)	0.00001 (0.000002–0.003)	0.0017 (0.0003–0.18)	0.021 (0.006–0.07)

DI: Daily intake; ^a^Hazard quotient (HQ) = DI/TDI × 100%; ^b^Median (Min-Max).
