# Supplementary material for: Estimated Daily Intake and Cumulative Risk Assessment of Phthalates in the General Taiwanese after the 2011 DEHP Food Scandal
Source: Sci Rep. 2017 Mar 22;7:45009. doi: 10.1038/srep45009 (PMC5361203; doi:10.1038/srep45009)
Supplement: Supplementary Tables [file srep45009-s1.pdf]

Supplementary Information

Title: Estimated Daily Intake and Cumulative Risk Assessment of Phthalates in the General Taiwanese after the 2011 DEHP Food Scandal

Jung-Wei Chang[1, +], Ching-Chang Lee[1,2, +], Wen-Harn Pan[3,4], Wei-Chun Chou[5], Han-Bin Huang[6], Hung-Che Chiang[5], Po-Chin Huang[5,7,8,*]

[1]Research Center for Environmental Trace Toxic Substances, National Cheng Kung University, Tainan, Taiwan;

[2]Department of Environmental and Occupational Health, College of Medicine, National Cheng Kung University, Tainan, Taiwan;

[3]Institute of Biomedical Sciences, Academia Sinica, Taipei, Taiwan;

[4]Division of Preventive Medicine and Health Service Research, Institute of Population Health Sciences, National Health Research Institutes, Miaoli, Taiwan;

[5]National Environmental Health Research Center, National Institute of Environmental Health Sciences, National Health Research Institutes, Miaoli, Taiwan;

[6]School of Public Health, National Defense Medical Center, Taipei, Taiwan;

[7]Research Center for Environmental Medicine, Kaohsiung Medical University, Kaohsiung, Taiwan;

[8]Department of Safety, Health and Environmental Engineering, National United University, Miaoli, Taiwan.

[+] these authors contributed equally to this work

Corresponding author (*): Dr. Po-Chin Huang, PhD,

National Environmental Health Research Center, National Institute of Environmental Health Sciences, National Health Research Institutes, Miaoli, Taiwan; E-mail: pchuang@nhri.org.tw

Supplemental Table 1. The Molecular weights, urinary excretion fractions and reference values of exposure to six phthalates by USEPA, EFSA and WHO.

Parent phthalate Compounds	Phthalate monoester metabolite	Molecular weight of diester parent compound, g/mole (MW_{parent})	Molecular weight of metabolite, g/mole ($MW_{metabolite}$)	Urinary excretion fraction (FUE, %)[a]	Carcinogenicity[b]	Endpoint	RfD[b]	Endpoint	TDI[c]
DEP	MEP	222.2	194	69	D	Organ weight	800	Organ weight	500[d]
DnBP	MBP	278.4	222.2	69	D	Increased mortality	100	Reproductive effect	10
BBzP	MBzP	312.4	256.3	73	C	Liver effect	200	Reproductive effect	500
DiNP	MiNP	418.6	292	2.1	-	-	-	Liver effect	150
DiBP	MiBP	278.4	222.24	69				Reproductive effect	10[e]
DEHP	MEHP	390.56	278.34	5.9	B2	Liver weight	20	Reproductive effect	50
	MEHHP	390.56	294.35	23.3					
	MEOHP	390.56	292.33	15.0					
	MECPP	390.56	308.33	18.5					
	MCMHP	390.56	312.3	4.2					

[a]The FUEs are taken from the following studies: DEHP [1, 2]; DBP [3]; BBzP [3]; DiNP [4]; DiBP [3];

[b]USEPA (US Environmental Protection Agency) & IRIS (Integrated Risk Information System), Available: http://www.epa.gov/iris/subst; RfD (Reference Dose) (μg/kg-bw/day); [c]EFSA (European Food Safety Authority), 2005; TDI (Tolerable Daily Intake) (μg/kg-bw/day);

[d]WHO (World Health Organization), 2003; [e]DiBP has been reported to reduce fetal testosterone production with similar potency to DnBP.

1. Koch, H.M., Bolt, H.M. & Angerer, J. Di(2-ethylhexyl)phthalate (DEHP) metabolites in human urine and serum after a single oral dose of deuterium-labelled DEHP. *Arch Toxicol* **78**, 123-130 (2004).
2. Koch, H.M., Bolt, H.M., Preuss, R. & Angerer, J. New metabolites of di(2-ethylhexyl)phthalate (DEHP) in human urine and serum after single oral doses of deuterium-labelled DEHP. *Arch Toxicol* **79**, 367-376 (2005).
3. Anderson, W.A.C., Castle, L., Scotter, M.J., Massey, R.C. & Springall, C. A biomarker approach to measuring human dietary exposure to certain phthalate diesters. *Food Addit Contam* **18**, 1068-1074 (2001).
4. Koch, H.M. & Angerer, J. Di-iso-nonylphthalate (DINP) metabolites in human urine after a single oral dose of deuterium-labelled DINP. *Int J Hyg Environ Health* **210**, 9-19 (2007).

Supplemental Table 2. PCA of phthalate metabolites in urine sample.

Compounds	Rotated Pattern loadings [a]											
	Male minors			Male Adults			Female minors			Female Adults		
	FA1	FA2	FA3	FA1	FA2	FA3	FA1	FA2	FA3	FA1	FA2	FA3
MEP	0.11	0.07	0.86	-0.02	0.15	-0.39	-0.06	-0.42	-0.02	0.06	0.09	-0.52
MiBP	-0.02	0.75	0.42	-0.05	0.19	0.67	0.03	0.64	0.48	0.32	0.15	0.55
MnBP	0.16	0.70	0.06	-0.08	0.77	-0.01	0.49	0.24	0.39	0.02	0.09	0.74
MBzP	0.01	0.53	-0.13	0.14	0.02	0.68	0.07	-0.07	0.92	0.30	0.81	0.00
MEHP	0.84	0.00	-0.11	0.25	0.46	0.26	0.62	0.43	-0.16	0.72	0.21	0.16
MEHHP	0.91	0.22	0.05	0.94	0.06	0.07	0.77	0.08	0.01	0.86	0.41	0.09
MEOHP	0.76	0.51	-0.11	0.94	0.06	0.14	0.90	0.09	0.01	0.85	0.42	0.08
MECPP	0.89	0.10	0.12	0.96	0.05	0.06	0.86	0.06	0.25	0.95	0.08	-0.03
MCMHP	0.87	-0.05	0.02	0.92	0.02	-0.03	0.78	-0.05	0.06	0.92	-0.08	-0.01
MiNP	0.24	0.48	-0.47	0.07	0.68	-0.09	0.04	0.79	-0.10	0.06	0.83	0.04
Extraction sums of squared loadings												
Eigenvalues	3.78	1.89	1.20	3.66	1.34	1.16	3.39	1.47	1.33	3.92	1.79	1.17
% of Variance	37.79	18.88	11.98	36.58	13.39	11.63	33.92	14.70	13.30	39.19	17.91	11.67
Cumulative %	37.79	56.67	68.65	36.58	49.98	61.60	33.92	48.62	61.92	39.19	57.10	68.76

[a] Rotated method: Varimax

Supplemental Table 3. Pearson correlations coefficients between the urinary phthalate metabolites and daily intake of phthalates in different relationship.

	Phthalate metabolite[a] (μmol/g cre)			Daily intake of phthalate[a] (μg/kg/day)	
	Parents (n = 62)	Couple/siblings (n = 37)		Parents (n = 62)	Couple/siblings (n = 37)
MMP	**0.323***	0.213	DMP	**0.352****	0.215
MiBP	**0.609****	**0.701****	DiBP	**0.649****	**0.746****
MnBP	**0.339****	**0.370***	DnBP	**0.371****	**0.446****
MBzP	0.192	0.190	BBzP	0.195	0.135
MEHP	**0.400****	**0.319**$^{\#}$			
MEHHP	**0.417****	**0.411***			
MEOHP	-0.040	**0.592****			
MECPP	**0.406****	**0.505****			
MCMHP	**0.333****	**0.590****			
MiNP	0.199	-0.056	DINP	0.162	-0.138
MEP	0.135	0.325*	DEP	0.103	**0.423****
$\Sigma DBP_{(i+n)}$	**0.359****	0.141	$\Sigma DBP_{(i+n)}$	**0.427****	**0.282**$^{\#}$
ΣDEHP	0.197	**0.489****	ΣDEHP	0.162	**0.335***

$\Sigma DBP_{(i+n)}$: sum of MnBP and MiBP; ΣDEHP: sum of MEHP, MEHHP, MEOHP, MECPP, and MECPP.

$^{\#}0.05 < p < 0.1$, $^{*}p < 0.05$, $^{**}p < 0.01$.

[a]Phthalate metabolite levels and daily intake of phthalate dose were natural log-transformed.
